# Prognostic Nomograms and Scoring System: Novel Approaches to Forecast Overall Survival and Cancer‐Specific Survival in Patients With Testicular Cancer

**DOI:** 10.1002/cam4.71515

**Published:** 2026-02-20

**Authors:** Xiaoqi Huang, Lian Zhu, Mengjie Sun, Yuan Zhou, Yingman Ding, Changming Lin

**Affiliations:** ^1^ Department of Urology Surgery The People's Hospital of Xuancheng City Xuanzhou Xuancheng China; ^2^ Wannan Medical College Yijiang China; ^3^ Department of Nephrology The People's Hospital of Xuancheng City Xuanzhou Xuancheng China; ^4^ Department of Urology Surgery Huaian 82 Hospital Qinghe Huaian China

**Keywords:** nomogram, pathological type, prognostic tool, SEER, significant predictors, survival, testicular cancer

## Abstract

**Objectives:**

To develop efficacious assessment tools to individualize the evaluation of overall survival (OS) and cancer‐specific survival (CSS) in patients with testicular cancer.

**Methods:**

A total of 30,689 patients diagnosed with testicular cancer between 2004 and 2021 were selected from the Surveillance, Epidemiology, and End Results database. The study population was randomly divided into a training cohort and a validation cohort. Univariate and multivariate Cox analyses were conducted to identify significant predictors, which were subsequently utilized to construct nomograms for predicting 1‐, 3‐, 5‐, and 10‐year OS and CSS. The predictive performance of the nomograms underwent internal and external testing with the application of the concordance index (C‐index), receiver operating characteristic curves, and calibration curves. We developed a prognostic scoring system based on the coefficients within the Cox models for each subgroup.

**Results:**

The significant predictors included age, race, marital status, TNM stage, radiation, chemotherapy, surgery and pathology. Age emerged as the most potent factor associated with overall and cancer‐specific death (≥ 60 vs. < 30 years old: HR = 12.19 for OS and HR = 5.94 for CSS, *p* < 0.001). Among all pathological subtypes, choriocarcinoma exhibited the worst OS and CSS (reference seminoma: HR = 2.79 for OS and HR = 5.02 for CSS, *p* < 0.001). The favorable internal validation (C‐index: 0.799 for OS and 0.859 for CSS; area under the curve = 0.773–0.892), external validation (C‐index: 0.784 for OS and 0.867 for CSS) and calibration curves indicated the nomograms possessed good predictive ability. We developed a prognostic scoring system for the first time, which is more accurate than the traditional TNM system in evaluating patients' survival outcomes.

**Conclusion:**

The prognostic nomograms and scoring systems are capable of effectively evaluating the 1‐, 3‐, 5‐, and 10‐year OS and CSS of testicular cancer patients and providing a reliable tool for optimizing clinical treatment decisions and follow‐up management.

**Abbreviations**
AUC: area under the curve
CI: confidence interval
C‐index: concordance index
CSS: cancer‐specific survival
HR: hazard ratio
OS: overall survival
PSM: propensity score matching
ROC: receiver operating characteristic
SEER: Surveillance Epidemiology and End Results
TNM: tumor‐node‐metastasis
VIF: variance inflation factor

## 1 Introduction

Testicular cancer primarily originates in the gonads and accounts for approximately 1% of male tumors, but it is the most common malignant tumor diagnosed in men aged 15–40 [1]. A significant shift in the age at diagnosis of testicular cancer has been observed, with the mean age increasing from 28 years pre‐1990 to 36 years in 2010 [2]. The incidence rate of testicular cancer is gradually rising. The global age‐standardized incidence rate of testicular cancer increased from 1.9 in 1990 to 2.8 in 2019 [3]. According to the statistics from the International Agency for Research on Cancer in 2022, there were approximately 72,031 newly diagnosed cases and 9056 deaths attributable to testicular cancer worldwide [4].

As the “gold standard” for tumor staging, the tumor‐node‐metastasis (TNM) staging system has long served as the cornerstone of prognostic stratification and initial treatment decision‐making in testicular cancer. However, the TNM system, which primarily relies on anatomical metrics, inherently fails to capture the unique biological heterogeneity that characterizes testicular cancer. Testicular germ cell tumors constitute over 95% of testicular malignancies and are classified into seminomatous germ cell tumors and nonseminomatous germ cell tumors [1]. Nonseminomas primarily include embryonal carcinoma, choriocarcinoma, yolk sac tumor, teratoma, etc., and are more invasive than seminomatous germ cell tumors [5].

Meanwhile, key clinical characteristics of patients, including age at diagnosis, race, marital status, and treatment modality, have been demonstrated to exert a significant impact on survival outcomes in patients with testicular cancer. Several studies have indicated that young testicular cancer patients exhibit notable survival differences compared with older counterparts with identical tumor stage and pathology [6, 7]. A long‐term follow‐up study involving 27,948 testicular cancer patients demonstrated that different races, marital status, and treatment methods exert a significant influence on the survival of testicular cancer patients [8]. Therefore, sole reliance on the traditional TNM staging system for survival assessment lacks sufficient accuracy, necessitating the development of more refined and personalized predictive tools for patients with testicular cancer.

Nomogram, a method for visually mapping multiple significant predictors, has been extensively applied in predicting the development of various diseases and the survival of cancer patients in recent years [9, 10]. Thus, this study intends to construct effective assessment tools based on a comprehensive dataset, enabling personalized survival evaluation for testicular cancer patients. The outcomes of our research may facilitate clinicians in formulating personalized treatment regimens and follow‐up strategies.

## 2 Materials and Methods

### 2.1 Data Source

We retrospectively analyzed patients diagnosed with testicular cancer during the period from 2004 to 2021 in the Surveillance, Epidemiology and End Results (SEER) database (SEER 21 Regs Data, based on the submission in November 2021, version 8.4.4). The SEER database represents the largest cancer registry and encompasses approximately 28% of the cancer registries within the United States [11].

The SEER database is a publicly accessible and identifiable database. Consequently, this study did not require informed consent from patients or permission from ethics committees.

### 2.2 Study Population

We obtained the available information including age, race, marital status, T stage (tumor invasion), N stage (lymph node metastasis), M stage (distant metastasis), radiation, chemotherapy, surgery, pathological type, overall survival (OS) status, cancer‐specific survival (CSS) status and survival time from SEER database. Patients with missing information mentioned above, non‐pathological diagnoses, or a history of other cancers were excluded. Due to the minuscule proportion of testicular cancer patients under the age of 15, accounting for less than 1%, they were not included in the study. Upon adhering to the above selection criteria, we observed that excluding patients with TX stage resulted in only 59 cases without surgical treatment. Therefore, we retained patients with TX stage.

### 2.3 Statistical Analysis

We randomly divided testicular cancer patients diagnosed in the year of 2005, 2009, 2010, 2014, and 2019 as the validation cohort, while the remaining patients were designated as the training cohort. Univariate and multivariate Cox regression analyses were performed in the training cohort, and variables exhibiting *p* values less than 0.05 were identified as significant predictors for establishing nomograms to predict 1‐, 3‐, 5‐, and 10‐year OS and CSS in patients with testicular cancer. Collinearity among clinical variables and final radiomics features was assessed through variance inflation factor (VIF) analysis. Concordance index (C‐index), receiver operating characteristic (ROC) curves, and calibration curves were utilized to evaluate the internal and external prediction accuracy of the nomograms. Bootstrapping validation, involving 1000 bootstrap resamples, was applied to the nonadherence nomograms. The C‐index and the area under the curve (AUC) values of 100% signify a flawless prediction, whereas values exceeding 0.7 indicate that the model is efficacious in prediction [12]. The closer the predicted curve is to the observed curve in the calibration curve, the more accurate the nomogram prognosis will be. We established nomograms entirely based on the TNM classification system to predict OS and CSS, thereby quantifying the incremental improvement of the proposed prediction model relative to existing criteria. Additionally, a prognostic scoring system for OS and CSS in testicular cancer patients was first developed, with parameters based on the coefficients from each subgroup's Cox model. The maximum scores of all variables in the prognostic scoring system were summed to a total of 100 points, and patients were stratified into four prognostic grades according to their total cumulative scores. Kaplan–Meier curves were utilized to compare OS and CSS between the traditional TNM staging system and the proposed prognostic scoring system in patients with testicular cancer. All statistical analyses and figure generation were conducted using R software (version 4.4.1), with statistical significance defined as *p* < 0.05.

## 3 Results

### 3.1 Characteristics of the Study Patients

**FIGURE 1:**
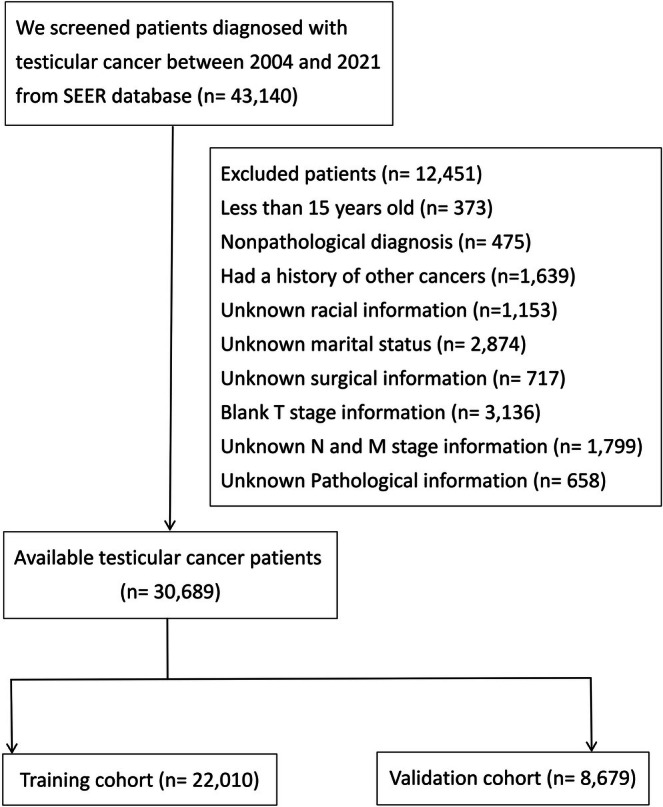
Flow chart for screening testicular cancer patients from the SEER Database.

A total of 30,689 testicular cancer patients were selected for analysis based on the selection criteria, with 22,010 assigned to the training cohort and 8679 to the validation cohort (Figure 1). The patients in both the training cohort and validation cohort exhibited similar distributions and 1‐, 3‐, 5‐, and 10‐year survival rates (OS: training cohort vs. validation cohort, 98.1%, 96.0%, 94.9%, and 92.6% vs. 98.3%, 96.2%, 95.2%, and 92.9%, respectively; CSS: training cohort vs. validation cohort, 98.7%, 97.2%, 96.8%, and 96.3% vs. 98.7%, 97.3%, 97.0%, and 96.5%, respectively). The baseline characteristics of testicular cancer patients are presented in Table 1.

**TABLE 1:** Baseline characteristics of patients with testicular cancer (*n* = 30,689, 2004–2021).

Patient characteristics	Training cohort		Validation cohort	
	(*n* = 22,010)		(*n* = 8679)	
	No. of patients	%	No. of patients	%
**Age**				
< 30	8570	38.9	3412	39.3
31~44	9487	43.1	3608	41.6
45~59	3323	15.1	1388	16.0
≥ 60	630	2.9	271	3.1
**Race**				
White	20,156	91.6	7970	91.8
Black	581	2.6	220	2.5
Other	1273	5.8	489	5.7
**Marital status**				
Married	9518	43.2	3910	45.1
Single	11,382	51.7	4262	49.1
Div/Sep/Wid	1110	5.1	507	5.8
**T (tumor invasion)**				
T1	14,296	64.9	5697	65.7
T2	6064	27.6	2347	27.0
T3	978	4.4	411	4.7
T4	108	0.5	36	0.4
TX	564	2.6	188	2.2
**N (lymph node metastasis)**				
No	17,109	77.7	6780	78.1
Yes	4901	22.3	1899	21.9
**M (distant metastasis)**				
No	19,590	89.0	7766	89.5
Yes	2420	11.0	913	10.5
**Surgery**				
No	257	1.2	107	1.2
Radical orchiectomy	21,233	96.4	8364	96.4
Partial or simple orchiectomy	520	2.4	208	2.4
**Radiation**				
Yes	3127	14.2	1487	17.1
None/unknown	18,883	85.8	7192	82.9
**Chemotherapy**				
Yes	8176	37.1	3254	37.5
None/unknown	13,834	62.9	5425	62.5
**Pathology**				
Seminoma	12,020	54.6	4829	55.6
Mixed germ cell tumor	6721	30.5	2467	28.5
Teratoma	616	2.8	281	3.2
Embryonal carcinoma	1744	7.9	738	8.5
Choriocarcinoma	467	2.1	174	2.0
Yolk sac tumor	208	1.0	88	1.0
Other types	234	1.1	102	1.2
**Death status**				
Alive	20,602	93.6	8072	93.0
Death	1408	6.4	607	7.0

*Note:* Patients diagnosed in the year of 2005, 2009, 2010, 2014 and 2019 as the validation cohort, and used the residual patients as the training cohort. We used the training cohort to develop nomograms and internal validation and used the validation cohort for external validation.

Abbreviation: Div/Sep/Wid, divorced, separated, or widowed.

### 3.2 Screening of Significant Predictors

Variables with *p* < 0.05 in univariate and multivariate Cox regression analyses were identified as significant prognostic factors. As shown in Table 2, the significant predictors of OS and CSS in testicular cancer patients included age, race, marital status, T stage, N stage, M stage, radiation therapy, chemotherapy, surgical treatment, and pathological type. The multivariate regression analysis demonstrated that age and M stage were most strongly associated with overall death (≥ 60 vs. < 30 years old: HR = 12.19; 95% CI = 9.83–15.13; M1 vs. M0: HR = 4.10; 95% CI = 3.53–4.75; *p* < 0.001, respectively) and cancer‐specific death (≥ 60 vs. < 30 years old: HR = 5.94; 95% CI = 4.06–8.69; M1 vs. M0: HR = 5.86; 95% CI = 4.77–7.21; *p* < 0.001 for both) among testicular cancer patients. Of all pathological subtypes, choriocarcinoma demonstrated the poorest prognosis in terms of OS and CSS (reference seminoma: OS HR = 2.79; 95% CI = 2.20–3.51; CSS HR = 5.02; 95% CI = 3.75–6.73, *p* < 0.001 for all). All 10 independent variables had variance inflation factor (VIF) values within an acceptable range: age (1.142), race (1.001), marital status (1.092), T stage (1.264), N stage (1.449), M stage (1.353), radiation (1.122), chemotherapy (1.619), surgery (1.034), and pathological type (1.219). This indicates that no significant multicollinearity existed among the independent variables, ensuring the stability of subsequent model parameter estimates.

**TABLE 2:** The univariate and multivariate Cox regression analyses for overall survival (OS) and cancer‐specific survival (CSS) of patients with testicular cancer in the training cohort.

Patient characteristics	OS			CSS		
	Univariate	Multivariable		Univariate	Multivariable	
	*p*	HR (95% CI)	*p*	*p*	HR (95% CI)	*p*
**Age**	< 0.001			< 0.001		
< 30		Reference			Reference	
31~44		1.78 (1.55–2.05)	< 0.001		1.57 (1.30–1.88)	< 0.001
45~59		3.76 (3.19–4.44)	< 0.001		2.71 (2.13–3.45)	< 0.001
≥ 60		12.19 (9.83–15.13)	< 0.001		5.94 (4.06–8.69)	< 0.001
**Race**	0.001			0.029		
White		Reference			Reference	
Black		1.43 (1.11–1.85)	0.005		1.21 (0.82–1.78)	0.336
Other		1.31 (1.06–1.61)	0.014		1.39 (1.05–1.84)	0.023
**Marital status**	< 0.001			< 0.001		
Married		Reference			Reference	
Single		2.13 (1.87–2.43)	< 0.001		2.16 (1.77–2.63)	< 0.001
Div/Sep/Wid		2.07 (1.71–2.50)	< 0.001		1.75 (1.28–2.40)	< 0.001
**T (tumor invasion)**	< 0.001			0.003		
T1		Reference			Reference	
T2		1.05 (0.92–1.19)	0.505		1.14 (0.94–1.38)	0.190
T3		1.25 (1.04–1.51)	0.019		1.28 (1.00–1.62)	0.048
T4		1.66 (1.13–2.45)	0.011		2.03 (1.30–3.15)	0.002
TX		1.21 (0.91–1.61)	0.189		1.23 (0.87–1.74)	0.245
**N (regional lymph node)**	< 0.001			< 0.001		
No		Reference			Reference	
Yes		1.32 (1.16–1.51)	< 0.001		1.47 (1.24–1.75)	< 0.001
**M (metastasis)**	< 0.001			< 0.001		
No		Reference			Reference	
Yes		4.10 (3.53–4.75)	< 0.001		5.86 (4.77–7.21)	< 0.001
**Surgery**	0.010			0.003		
No		Reference			Reference	
Radical orchiectomy		0.50 (0.36–0.69)	< 0.001		0.45 (0.31–0.65)	< 0.001
Partial or simple orchiectomy		0.69 (0.45–1.04)	0.078		0.54 (0.31–0.95)	0.032
**Radiation**	< 0.001			< 0.001		
Yes		Reference			Reference	
None/Unknown		0.64 (0.55–0.76)	< 0.001		0.42 (0.34–0.54)	< 0.001
**Chemotherapy**	0.038			< 0.001		
Yes		Reference			Reference	
None/Unknown		0.85 (0.73–1.00)	0.045		0.56 (0.44–0.72)	< 0.001
**Pathology**	< 0.001			< 0.001		
Seminoma		Reference			Reference	
Mixed germ cell tumor		1.60 (1.38–1.85)	< 0.001		2.82 (2.25–3.53)	< 0.001
Teratoma		1.99 (1.50–2.65)	< 0.001		3.84 (2.64–5.58)	< 0.001
Embryonal carcinoma		1.32 (1.07–1.64)	0.010		2.05 (1.51–2.80)	< 0.001
Choriocarcinoma		2.79 (2.20–3.51)	< 0.001		5.02 (3.75–6.73)	< 0.001
Yolk sac tumor		2.60 (1.86–3.63)	< 0.001		3.62 (2.34–5.61)	< 0.001
Other types		1.54 (1.08–2.21)	0.018		2.13 (1.31–3.46)	0.002

Abbreviations: CI, confidence interval; Div/Sep/Wid, divorced, separated, or widowed; HR, hazard ratio.

### 3.3 Nomogram Establishment and Validation

**FIGURE 2:**
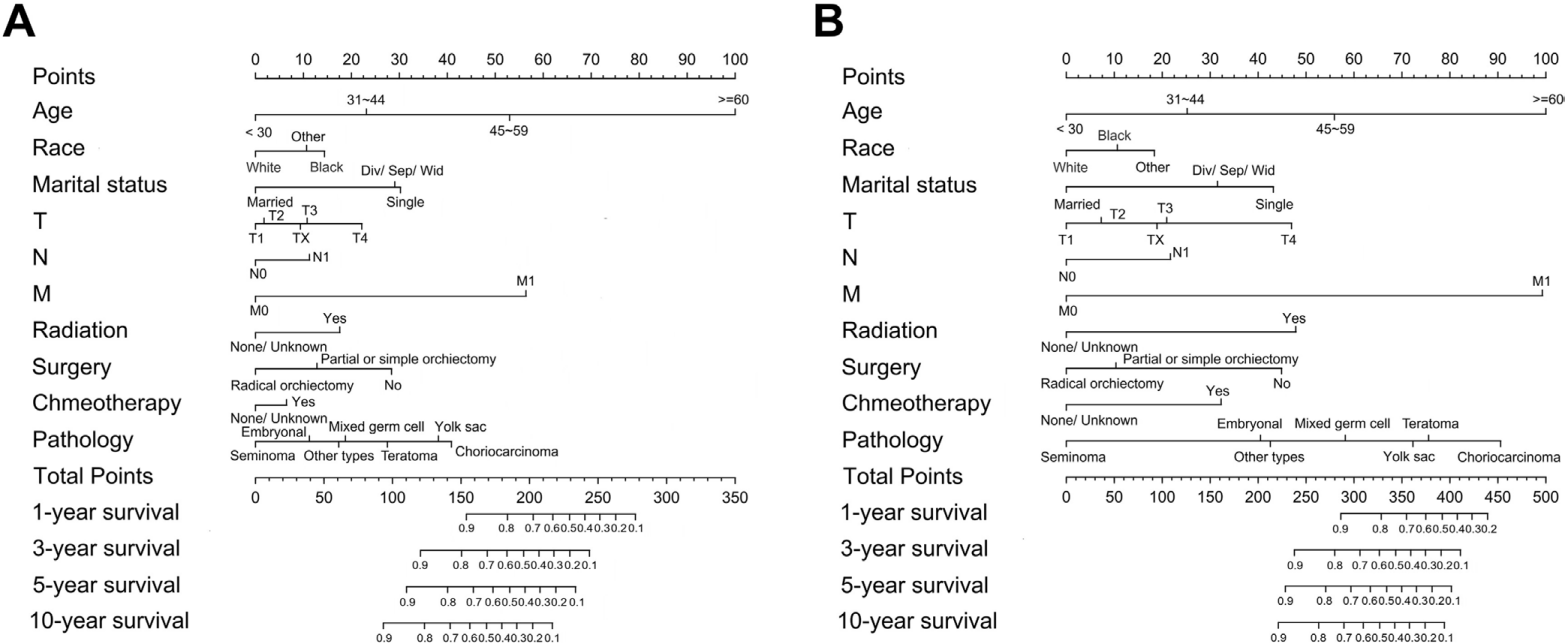
Nomograms designed to forecast the 1‐, 3‐, 5‐, and 10‐year OS (A) and CSS (B) of testicular cancer patients.

**FIGURE 3:**
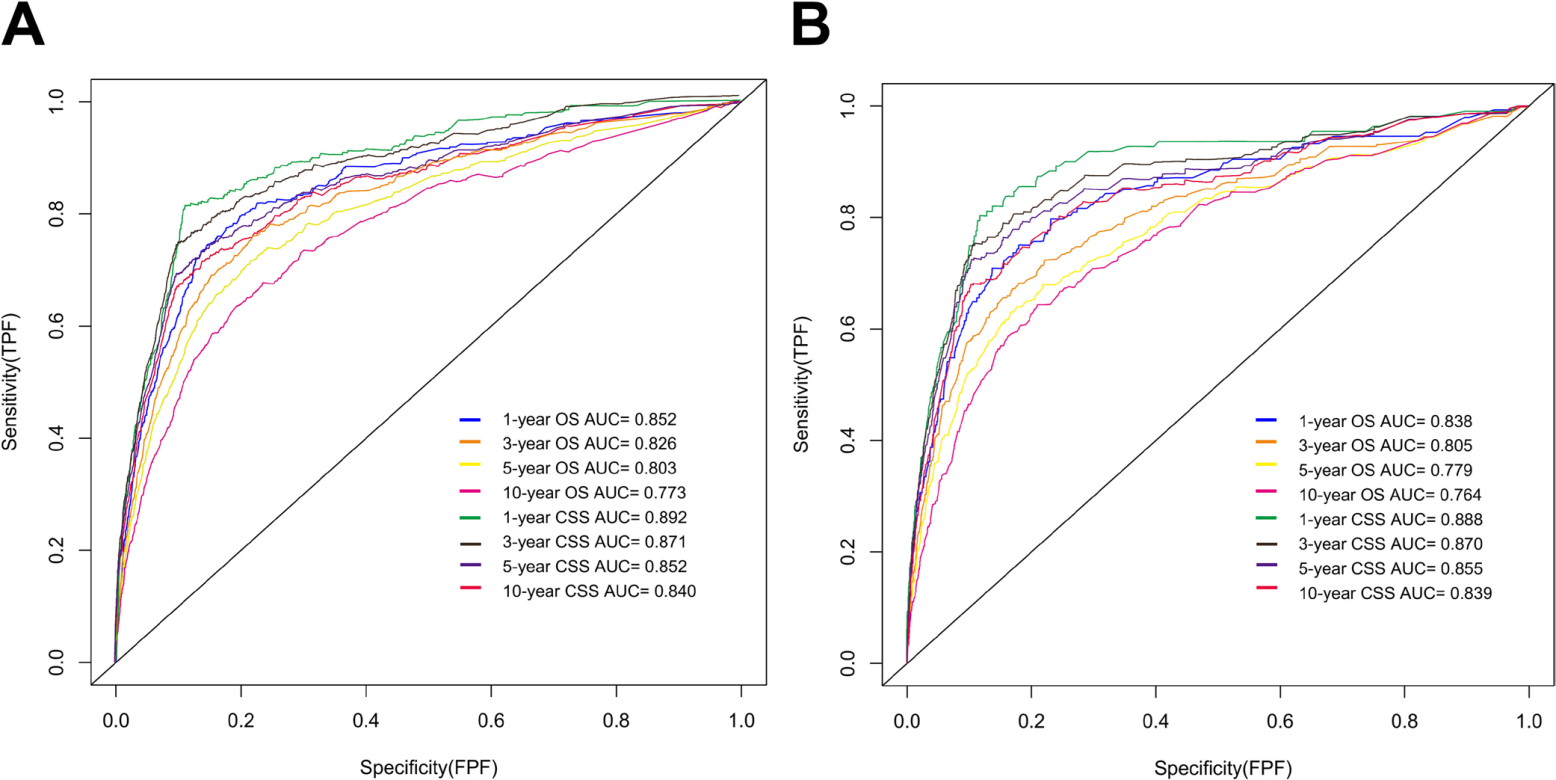
Receiver operating characteristic (ROC) curve to evaluate the ability of nomograms' prediction in the training cohort (A) and validation cohort (B).

**FIGURE 4:**
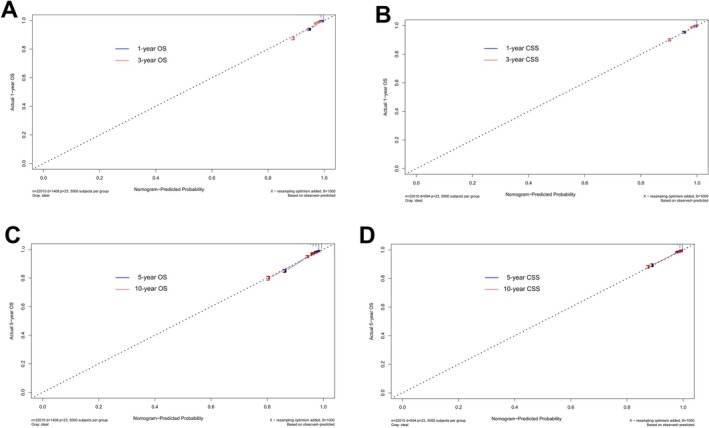
Calibration curves to test the ability of nomograms' prediction in the training cohort: (A) 1‐year and 3‐year OS; (B) 1‐year and 3‐year CSS; (C) 5‐year and 10‐year OS; (D) 5‐year and 10‐year CSS.

We utilized the significant predictors to construct survival prediction nomograms for 1‐, 3‐, 5‐, and 10‐year OS and CSS in testicular cancer patients (Figure 2). The sum of scores corresponding to each variable in the nomograms provided a preliminary estimate of survival outcomes. The favorable C‐index values (internal validation: OS C‐index = 0.799, 95% CI = 0.785–0.813; CSS C‐index = 0.859, 95% CI = 0.843–0.875; and external validation: OS C‐index = 0.784, 95% CI = 0.764–0.803; CSS C‐index = 0.867, 95% CI = 0.843–0.890) and area under the curve (AUC) values (internal validation for 1‐, 3‐, 5‐, and 10‐year OS: AUC = 0.852, 0.826, 0.803, 0.773 and CSS: AUC = 0.892, 0.871, 0.852, 0.840, respectively; external validation for 1‐, 3‐, 5‐, and 10‐year OS: AUC = 0.838, 0.805, 0.779, 0.764 and CSS: AUC = 0.888, 0.870, 0.855, 0.839, respectively) demonstrated that the nomograms possessed good predictive ability (Figure 3). The calibration curves showed satisfactory consistency between the nomogram‐predicted outcomes and actual observations in the training cohort (Figure 4).

### 3.4 Prognostic Scoring System

**FIGURE 5:**
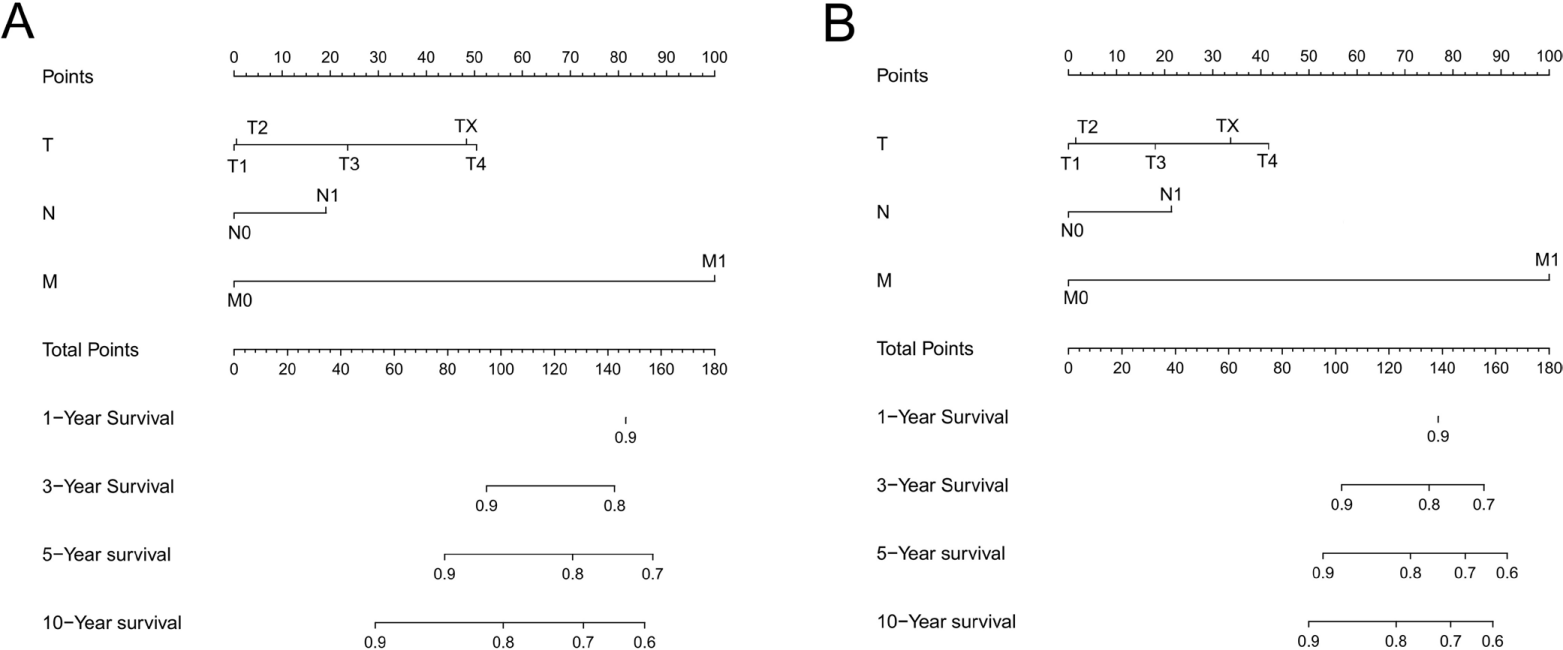
TNM staging‐based nomogram for predicting OS (A) and CSS (B) of testicular cancer patients.

**FIGURE 6:**
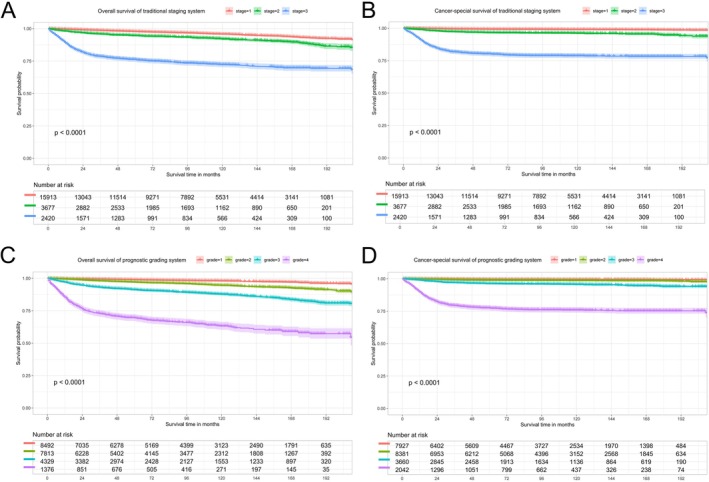
Kaplan–Meier curves to conduct a comparison of the OS and CSS among testicular cancer patients based on both the traditional TNM staging system and the prognostic scoring system: (A) OS for traditional TNM staging system; (B) CSS for traditional TNM staging system; (C) OS for prognostic scoring system; (D) CSS for prognostic scoring system.

For the purpose of quantifying the incremental improvement of the proposed prediction model over existing criteria, we developed nomograms that are entirely based on the TNM classification system to predict OS and CSS (C‐index for OS = 0.736, 95% CI = 0.743–0.790; C‐index for CSS = 0.839, 95% CI = 0.823–0.855). Nomograms integrated with multi‐dimensional clinical covariates (Figure 2) yield more precise and individualized predictions of patients' survival outcomes compared to those exclusively derived from the TNM staging system (Figure 5), with better alignment to real‐world clinical diagnostic and therapeutic paradigms. Drawing on the coefficients from Cox proportional hazards models for each subgroup, we established a prognostic scoring system to predict OS and CSS in patients with testicular cancer. The maximum score assigned to each variable within the scoring system was aggregated, yielding a total of 100 points. Higher scores within this scoring system correlated with a poorer survival prognosis (detailed assignment criteria for OS and CSS are summarized in Table 3 and Table 4, respectively). We categorized testicular cancer patients into four distinct prognostic strata based on their cumulative scores: Grade I (0–15), Grade II (16–25), Grade III (26–40), and Grade IV (41–100) points. Table 5 details the patient distribution and 1‐, 3‐, 5‐, and 10‐year OS and CSS rates for each stage (TNM) and prognostic grade of testicular cancer. Kaplan–Meier curves were utilized to compare OS and CSS among testicular cancer patients stratified by both the conventional TNM staging system and our novel prognostic scoring system. As illustrated in Figure 6, the latter yields more clear‐cut survival stratification, highlighting its superior discriminatory ability.

**TABLE 3:** Scores of significant predictors in the prognostic scoring system for overall death.

Patient characteristics	All‐cause death points	Patient characteristics	All‐cause death points
**Age**		**M (metastasis)**	
< 30	0	No	0
31 ~ 44	7	Yes	17
45 ~ 59	16	**Surgery**	
≥ 60	30	No	9
**Race**		Radical orchiectomy	0
White	0	Partial or simple orchiectomy	4
Black	4	**Radiation**	
Other	3	Yes	5
**Marital status**		None/Unknown	0
Married	0	**Chemotherapy**	
Single	9	Yes	3
Div/Sep/Wid	9	None/Unknown	0
**T (tumor invasion)**		**Pathology**	
T1	0	Seminoma	0
T2	1	Mixed germ cell tumor	6
T3	3	Teratoma	8
T4	7	Embryonal carcinoma	4
TX	3	Choriocarcinoma	12
**N (regional lymph node)**		Yolk sac tumor	11
No	0	Other types	5
Yes	4		

*Note:* The sum of the highest scores for each variable in the prognostic scoring system amounted to 100 points. A higher score within this system signified a poorer survival prognosis. We classed the survival of testicular cancer patients into four grades: 0~15 (Grade I), 16~25 points (Grade II), 26–40 points (Grade III), and 41~10 decimals (Grade IV).

**TABLE 4:** Scores of significant predictors in the prognostic scoring system for cancer‐specific death.

Patient characteristics	Cancer‐specific death points	Patient characteristics	Cancer‐specific death points
**Age**		**M (metastasis)**	
< 30	0	No	0
31~44	5	Yes	18
45~59	10	**Surgery**	
≥ 60	18	No	9
**Race**		Radical orchiectomy	0
White	0	Partial or simple orchiectomy	2
Black	2	**Radiation**	
Other	3	Yes	9
**Marital status**		None/Unknown	0
Married	0	**Chemotherapy**	
Single	8	Yes	6
Div/Sep/Wid	6	None/Unknown	0
**T (tumor invasion)**		**Pathology**	
T1	0	Seminoma	0
T2	1	Mixed germ cell tumor	11
T3	4	Teratoma	14
T4	9	Embryonal carcinoma	7
TX	3	Choriocarcinoma	16
**N (regional lymph node)**		Yolk sac tumor	13
No	0	Other types	8
Yes	4		

*Note:* The sum of the highest scores for each variable in the prognostic scoring system amounted to 100 points. A higher score within this system signified a poorer survival prognosis. We classed the survival of testicular cancer patients into four grades: 0~15 (Grade I), 16~25 points (Grade II), 26–40 points (Grade III), and 41~10 decimals (Grade IV).

**TABLE 5:** The comparison of overall survival (OS) and cancer‐specific survival (CSS) between testicular cancer patients stratified by the traditional TNM staging system and those stratified by the prognostic scoring system.

Stage (grade)	Survival time	Traditional staging system			Prognostic scoring system			
			OS	CSS	OS		CSS	
		Proportion (%)	Rate (%)	Rate (%)	Proportion (%)	Rate (%)	Proportion (%)	Rate (%)
I	3‐year	72.3	98.6	99.4	38.6	99.3	36.0	99.7
	5‐year		97.8	99.2		98.9		99.5
	10‐year		95.8	99.0		98		99.4
II	3‐year	16.7	95.8	97.2	35.5	98	38.1	99.1
	5‐year		94.6	96.6		97.1		98.8
	10‐year		92.0	96.1		94.5		98.5
III	1‐year	11.0	88.9	90.9	19.7	97.0	16.6	98.9
	3‐year		78.6	81.7		93.0		97.0
	5‐year		76.2	80.2		91.3		96.2
	10‐year		72.3	78.7		87.8		95.5
IV	1‐year	Unclassified			6.2	85.4	9.3	89.7
	3‐year					72.9		79.4
	5‐year					69.6		77.5
	10‐year					63.3		75.8

## 4 Discussion

Survival prediction for cancer patients represents a critical focus of oncology research, with medical data mining playing a pivotal role in guiding clinical practice. However, the traditional TNM system is constrained by its limited inclusion of prognostic factors, thereby failing to provide accurate individualized survival assessments for cancer patients. In this study, we developed nomograms to graphically visualize the predicted survival outcomes of testicular cancer patients based on complex mathematical algorithms. Internal and external validation confirmed that these nomograms effectively predicted 1‐, 3‐, 5‐, and 10‐year OS and CSS in testicular cancer patients. Key variables identified as significant predictors of testicular cancer prognosis included age, race, marital status, T stage, N stage, M stage, radiation therapy, chemotherapy, surgery, and pathological type, all of which markedly influenced patient survival outcomes. Multivariate regression analysis further indicated that age was the most strongly associated factor with both overall death (≥ 60 vs. < 30 years old: HR = 12.19; 95% CI = 9.83–15.13; *p* < 0.001) and cancer‐specific death (≥ 60 vs. < 30 years old: HR = 5.94; 95% CI = 4.06–8.69; *p* < 0.001) in testicular cancer patients. This stands in marked contrast to many other cancer types. In many other types of cancer survival prediction models, age had been demonstrated to be one of the most correlated factors with OS of patients, but this correlation is not particularly pronounced in CSS [9, 12, 13, 14]. Several biological and clinical mechanisms may explain this discrepancy. Younger testicular cancer patients typically exhibit more efficient tissue repair capacity, while aging correlates with delayed drug clearance and increased vulnerability to treatment‐related toxicity [15]. The increased toxicity of chemoradiotherapy in elderly patients aggravated cardiovascular and organ damage, increasing the risk of death [16, 17]. Additionally, prior studies indicated that older testicular cancer patients present with higher rates of advanced‐stage disease, faster progression, and increased recurrence incidence compared to younger cohorts [8, 18, 19]. The proportion of pathologic types with poor prognosis such as spermatocytic tumors, sex cord stromal tumors in older testicular cancer patients was significantly higher than that in younger patients [20]. To exclude the influence of factors such as cancer staging and pathology, we implemented propensity score matching (PSM) to test the impact of different ages on testicular cancer survival. The matched variables included race, marital status, TNM stage, surgery, radiotherapy and chemotherapy, and pathology, and the results in Table S1 showed that age differences still have a significant impact on OS and CSS of testicular cancer after PSM adjustment. Based on the confirmed prognostic impacts of age and M stage in this study, personalized treatment optimization is warranted in clinical practice. For young patients with stage M0 seminoma who desire fertility preservation, partial orchiectomy or simple orchiectomy may be selected to retain reproductive function while ensuring therapeutic efficacy. In contrast, elderly testicular cancer patients, characterized by poorer tumor biological behavior and a higher risk of occult metastasis, should be recommended radical orchiectomy, with retroperitoneal lymph node dissection added as appropriate. In elderly individuals diagnosed with high‐risk testicular cancer, follow‐up intervals should be shortened, and beyond standard tumor‐focused monitoring, treatment‐related adverse events (e.g., renal dysfunction, cardiovascular risks) should be assessed concurrently with timely, evidence‐based adjustments to intervention strategies.

In this study, the influence of several other variables on the survival of testicular cancer patients was in concordance with previous researches. White men may have the highest global incidence of testicular cancer, a pattern potentially attributed to their higher estradiol‐to‐testosterone ratio, yet they also demonstrate the highest survival rates; notably, non‐white patients with testicular cancer face a 1.69‐fold greater risk of death compared to white counterparts, a disparity that may be linked to the differences in healthcare services and diagnostic treatments [21]. Unmarried patients with testicular cancer have a risk of death nearly twice that of married patients, and this disparity is closely associated with the impact of multidimensional psychosocial factors throughout the entire disease course, specifically encompassing key links such as disease detection, treatment implementation, treatment adherence, treatment decision‐making, and post‐treatment monitoring or follow‐up adherence [22]. Clinical decisions for testicular cancer should be tailored to these findings: optimizing access to diagnosis and treatment resources based on race, enhancing psychosocial support stratified by marital status, and integrating these considerations into stratified assessment and whole‐course management of the disease. Relative to non‐seminomatous germ cell tumors, seminomas display both lower invasiveness and higher survival rates, and their survival outcomes demonstrate a more notable improvement as time progresses [4, 23, 24]. Our results were consistent with those studies and further demonstrated that patients with choriocarcinoma might have the worst OS and CSS. Additionally, this study indicates that a more comprehensive surgical treatment constituted a protective factor for the survival of testicular cancer patients. The radical surgery remains the most effective treatment option for solid tumors [25]. Currently, there remains a lack of consensus regarding the stage and dosage of adjuvant therapy for testicular cancer. Our results showed that patients who underwent radiotherapy and chemotherapy exhibited lower survival outcomes in comparison with those who did not. Testicular cancer is generally associated with a favorable prognosis, with the long‐term survival rate reaching 97% [26]. Specific types of testicular cancer demonstrate sensitivity to radiotherapy or chemotherapy, and the purpose of adjuvant radiotherapy or chemotherapy is to diminish the recurrence of testicular cancer and enhance patient survival. However, radiation and chemotherapy may impose an additional burden on organ function and increase the risk of developing a second malignancy and late complications [27, 28]. Previous research indicated that radiotherapy and chemotherapy increase the risk of second malignancy in testicular cancer, with a 2.6‐fold risk following radiotherapy and a 2.1‐fold risk following chemotherapy [28]. The lack of precise information on chemotherapy and radiotherapy in the SEER database complicates further analysis, requiring additional research and efforts to develop more effective treatment decisions based on the pathology types, tumor staging, and physical conditions of testicular cancer patients in clinical practice.

We developed a prognostic scoring system for the first time by leveraging the clinicopathological information to evaluate the survival of testicular cancer patients. The total score of the prognostic scoring system is 100 points, encompassing key clinical and pathological variables including patient age, race, marital status, TNM stage, treatment modalities (radiotherapy, chemotherapy, surgery), and pathological subtype. Within this system, TNM staging accounts for a maximum of 28 points in OS assessment and 31 points in CSS evaluation. Consequently, our prognostic scoring system enables more accurate assessment of patients' survival outcomes compared to the conventional TNM staging system alone. In clinical practice, the prognostic grade of individual patients can be determined simply by summing their respective scores based on the included variables. While the conventional TNM staging system categorizes testicular cancer patients into three stages (I, II, and III), our prognostic scoring system stratifies patients into four prognostic grades with a more comprehensive set of patient‐related variables. Comparative analysis with the traditional TNM staging system demonstrates that our prognostic scoring system yields more distinct survival stratification. Both the prognostic nomogram and scoring system can provide patients with intuitive initial survival expectations, based on which clinicians and patients can collaboratively formulate optimal treatment decisions. However, our prognostic models cannot completely replace clinical judgment. Clinicians need to strike a balance between individual distinctions and other factors, such as the concomitant diseases and physical conditions of patients in the decision‐making process. While this study is not the first prognostic nomogram model for testicular cancer patients [29, 30], it presents several advantages over previous models. Firstly, our data are derived from the latest SEER database and include follow‐ups until November 2021, owing to the updated dataset, which provided a longer follow‐up period for patients; we were able to add more precise 10‐year OS and CSS prediction for testicular cancer. Secondly, this study included surgical regimens, treatment information, and data covering all pathological types of testicular cancer, thereby generating a more comprehensive and less biased nomogram. Thirdly, our findings highlight that age is likely the factor most strongly associated with overall death and cancer‐specific death in patients with testicular cancer. Finally, we are the first to develop a prognostic scoring system that stratifies testicular cancer patients into four prognostic grades. Patients with higher grades should receive more intensive treatment and closer monitoring.

However, our research inevitably entails several limitations. Firstly, the unavailability of specific information, including patients' lifestyle, socioeconomic status, genetic background, cancer recurrence status, details of radiotherapy/chemotherapy regimens, and treatment centralization [31], may introduce bias into our results. Secondly, cohort studies possess inescapable drawbacks, such as sample selection bias and the effect of confounding factors. Finally, this study is essentially retrospective in nature and requires more prospective studies to validate our results.

## 5 Conclusions

This study developed nomograms and a prognostic scoring system for testicular cancer patients through a comprehensive analysis of a large cohort of 30,689 individuals. These tools integrate 10 key clinical and pathological variables, including age, race, marital status, TNM stage, radiotherapy, chemotherapy, surgery, and pathological type, and demonstrate robust predictive performance for 1‐, 3‐, 5‐, and 10‐year OS and CSS, as validated both internally and externally. Among the integrated variables, age emerged as the most critical prognostic factor for both OS and CSS, while choriocarcinoma exhibits the poorest survival outcomes among all pathological subtypes. Compared with the conventional TNM staging system, the proposed scoring system enables more distinct risk stratification, facilitating rapid identification of high‐risk patients. Our findings provide clinicians with a practical tool to accurately assess prognosis, guide individualized treatment strategies, and optimize risk‐adapted management of testicular cancer patients.

## Author Contributions

**Xiaoqi Huang:** formal analysis, writing – original draft. **Lian Zhu:** formal analysis, data curation, modification and statistical analysis. **Mengjie Sun:** writing – original draft, supervision. **Yuan Zhou:** supervision, validation. **Yingman Ding:** validation. **Changming Lin:** conceptualization, writing – review and editing.

## Funding

This study was supported by the Scientific research project of Xuancheng People‘s Hospital (KY202501) and Project of the Science and Technology Department of Anhui Province (2025AHGXZK31380).

## Ethics Statement

The SEER database is characterized by being publicly accessible and allowing for identification of relevant information. Given these attributes, this study did not require obtaining informed consent from patients or seeking permission from ethics committees.

## Conflicts of Interest

The authors declare no conflicts of interest.

## Supporting information


**Table S1:** The comparison of overall death risk and all cancer‐specific death risk for age after PSM.

## Data Availability

The data that support the findings of this study are available from the corresponding author upon reasonable request.
