# Supplementary material for: Prognostic Nomograms and Scoring System: Novel Approaches to Forecast Overall Survival and Cancer‐Specific Survival in Patients With Testicular Cancer
Source: Cancer Med. 2026 Feb 20;15(3):e71515. doi: 10.1002/cam4.71515 (PMC12927951; doi:10.1002/cam4.71515)
Supplement: Supplementary file 1 — Table S1: The comparison of overall death risk and all cancer‐specific death risk for age after PSM. [file CAM4-15-e71515-s001.docx]

| **Supplementary Table 1:** The comparison of overall death risk and all cancer-specific death risk for age after PSM. | | | | |
| --- | --- | --- | --- | --- |
| **Patient**  **Characteristics** | **OS** | | **CSS** | |
|  | **HR (95% CI)** | ***P* value** | **HR (95% CI)** | ***P* value** |
| Age |  | |  | |
| < 30 | Reference | | Reference | |
| 31~ 44 | 1.86(1.58-2.20) | <0.001 | 1.45(1.15-1.82) | <0.001 |
| 45~ 59 | 2.79(2.30-3.39) | <0.001 | 1.73(1.33-2.27) | <0.001 |
| >= 60 | 8.03(6.00-10.77) | <0.001 | 4.34(2.71-6.96) | <0.001 |
| **Abbreviations:** HR=hazard ratio, CI=confidence interval, PSM= propensity score matching. | | | | |
